# How to Make a Mother in Five Easy Steps

**DOI:** 10.1371/journal.pbio.0020359

**Published:** 2004-09-21

Assembling a complex structure like an automobile requires the tight coordination of hundreds of independent entities—parts must be shipped and arrive on time, workers with the right skills must be in the right place on the assembly line, four (not three, not five) wheels must be bolted into place just so. Overseeing the entire operation is a cadre of managers, whose ability to monitor and respond to changing conditions keeps the entire process moving forward on time and in step.[Fig pbio-0020359-g001]

**Figure pbio-0020359-g001:**
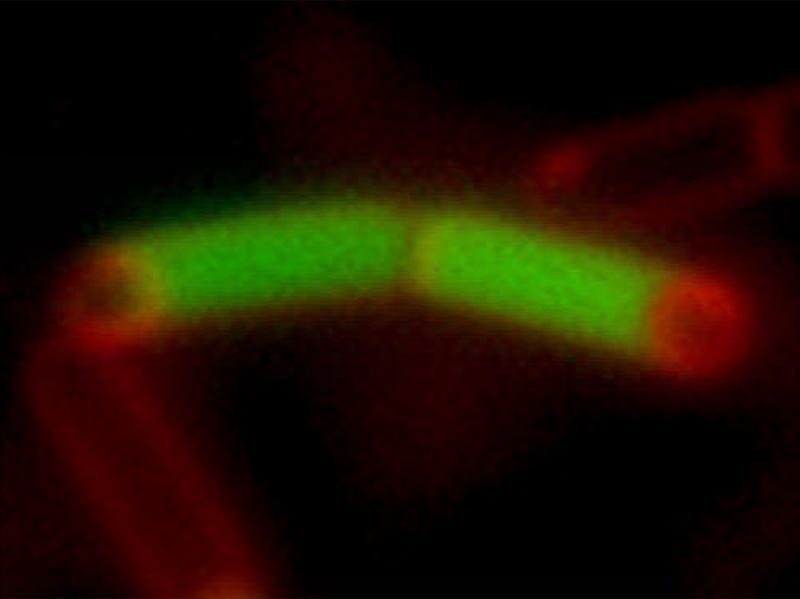
A pair of Bacillus subtilis sporangia, consisting of a large mother cell (green) and a forespore (red)

A living cell is orders of magnitude more complex, and yet has no omniscient manager at the helm. So how does a cell make anything happen on time, and equally important, keep everything from happening all at once? These central questions in developmental biology now have the outlines of an answer in one species, Bacillus subtilis. In this issue, Richard Losick and colleagues show that spore formation in this bacterium is ultimately governed by the temporal interactions of five genes, which together coordinate the activity of almost 400 others.

When conditions are right, B. subtilis divides to form two different cell types: one is a resistant spore, and the other is a mother cell, which engulfs the spore and surrounds it with a protective coat. Building on the large literature addressing the genetic events underlying mother-cell development, Losick et al. performed a variety of experiments to determine exactly which genes turned on and off when, and which genes controlled which others. Over the five-hour process of mother-cell development, they determined that 383 individual genes were activated, representing 9% of the bacterium's 4,106 genes.

The instigator of the entire process is a protein called sigmaE (σ^E^). Sigma factors, such as the sigma-E protein, bind to RNA polymerase, and in so doing, increase its affinity for, and therefore its ability to activate, other genes. Thus, sigma factors preferentially activate a specific set of genes. Sigma-E turns on 262 genes (which together compose its “regulon”), kick-starting a variety of processes in the early development of the mother cell.

Importantly, two of its targets, SpoIIID and GerR, are genes for DNA-binding proteins, which themselves modulate the expression of genes in the middle phase of development. Part of SpoIIID's portfolio is turning off transcription of a portion of the sigma-E regulon, and amplifying transcription of another portion. This type of control circuit, in which A leads to B, and then both A and B influence C, is called a feed-forward loop.

Among the joint targets for sigma-E and SpoIIID is another sigma factor, sigma-K (σ^K^). By generating the DNA-binding protein GerE, this factor begins a second feed-forward loop, and together, sigma-K and GerE activate the final set of genes needed for mother-cell development. In outline, the system looks like this: sigma-E → SpoIIID/GerR → sigma-K → GerE.

The consequence of all this activity is a series of transcriptional pulses, timed to supply proteins just as they are needed, and then turn off their production when the need passes. For instance, to form the multilayered coat around the spore, sigma-E turns on genes that form the bottom layer, or substratum; these are turned off by SpoIIID. Genes for outer layers, also turned on by sigma-E, are not turned off by SpoIIID, but instead by GerE. Sigma-K turns on genes which form the polysaccharide surface of the coat, which is needed later on.

The elucidation of this complex pattern of gene expression doesn't by any means answer every question about B. subtilis development, let alone development in more complex organisms. There is much still to be learned about how genes lower down in the hierarchy—the “middle managers”—do their jobs, and how the system is fine-tuned by environmental conditions. And while the general scheme of feed-forward loops and hierarchical control is likely to apply to multicellular, eukaryotic organisms, the details are certain to be different, and much more complex.

